# Reversible median nerve neuropathy and local muscle irritation resulting from blind removal attempts of etonogestrel contraceptive implant: a case report

**DOI:** 10.1186/s40834-023-00257-5

**Published:** 2023-12-01

**Authors:** Siraphat Fungtammasan, Natchanika Sinthuchai, Kawee Pataradool, Unnop Jaisamrarn, Somsook Santibenchakul

**Affiliations:** 1 Department of Obstetrics and Gynecology, Faculty of Medicine, Chulalongkorn University, Rama IV Road, Pathum Wan, Bangkok, Thailand; 2 Department of Epidemiology, Harvard T.H. Chan School of Public Health, 677 Huntington Avenue, Boston, MA USA; 3 Department of Orthopedics, Faculty of Medicine, Chulalongkorn University, Rama IV Road, Pathum Wan, Bangkok, Thailand; 4 Department of Obstetrics and Gynecology, King Chulalongkorn Memorial Hospital, Rama IV Road, Pathum Wan, Bangkok, Thailand

**Keywords:** Etonogestrel contraceptive implant, Deep insertion, Median nerve neuropathy, Difficult removal, Case report

## Abstract

Nexplanon is an etonogestrel contraceptive implant that comes with an applicator, making it easier to insert and remove. Complications related to insertion and removal procedures, such as neural-vascular injuries, are rare. We describe a case of reversible median nerve neuropathy and local muscle irritation resulting from blind removal attempts of an iatrogenically migrated implant. The patient presented with an unusual pain at the surgical site along with abnormal sensations and numbness in her left hand that worsened after blind attempts to remove the implant. Radiographs revealed that the rod was 3 cm from her insertion scar and deeply embedded in her left arm. The patient then underwent left arm exploration and implant removal under fluoroscopic guidance by an orthopedic surgeon. The rod was placed intramuscularly, adjacent to the median nerve under the basilic vein. The abnormal sensations and numbness in her left hand could be attributed to median nerve involvement, while the atypical pain at the surgical site could be a result of local irritation from the intramuscularly migrated implant from attempts at removal. The symptoms gradually resolved after surgery. This indicates that patients with impalpable contraceptive implants should be referred for implant removal by specialists familiar with the procedure to prevent further deterioration of adjacent structures from iatrogenic implant migration.

## Introduction

Since 2014, long-acting reversible contraception has been freely available to all adolescents in Thailand under the national healthcare program, consequently resulting in increased usage of contraceptive implantation. Nexplanon®, an etonogestrel contraceptive implant available since 2011, is a small, flexible, radiopaque rod that is pre-loaded in a disposable applicator [1]. The applicator was specially designed such that the implant could be easily inserted and removed if performed correctly. Complications of implant insertion and removal occur infrequently [2]. Case reports and case series have addressed the complications associated with insertion and removal procedures [3–7]. This case report will support healthcare providers regarding specialist referral if there are any difficulties/complications related to implant removal, such as impalpable contraceptive implants, abnormal sensations, or persistent pain at the implantation site or in the arm and hand on the same side, as removal-related complications are often anticipated with the aforementioned conditions [2, 8]. Blind removal attempts with impalpable contraceptive implants should be highly discouraged [2].

## Case presentation

**Fig. 1:**
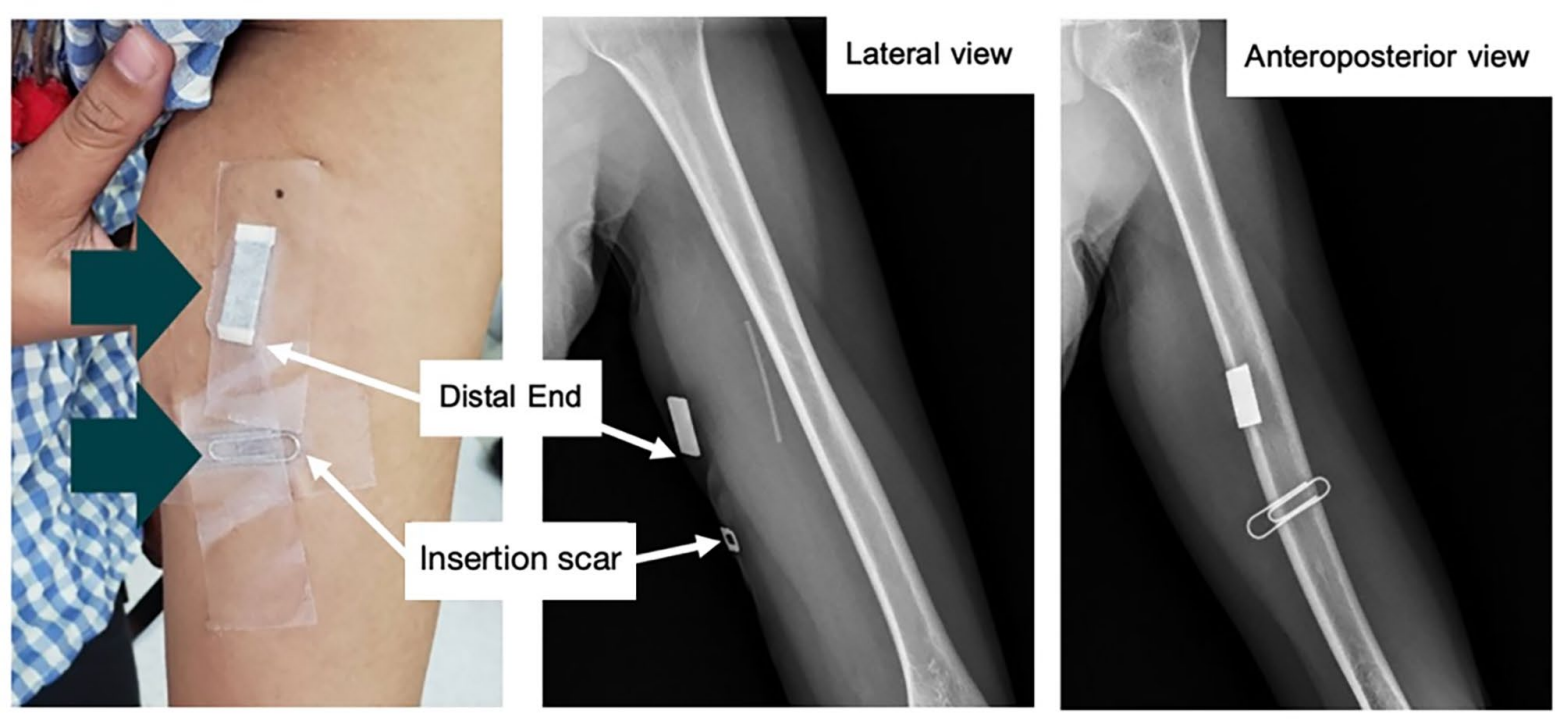
Antero-posterior and lateral radiographs of left upper arm revealed that the distal end of the implant was 3 cm distant from the insertion scar

**Fig. 2:**
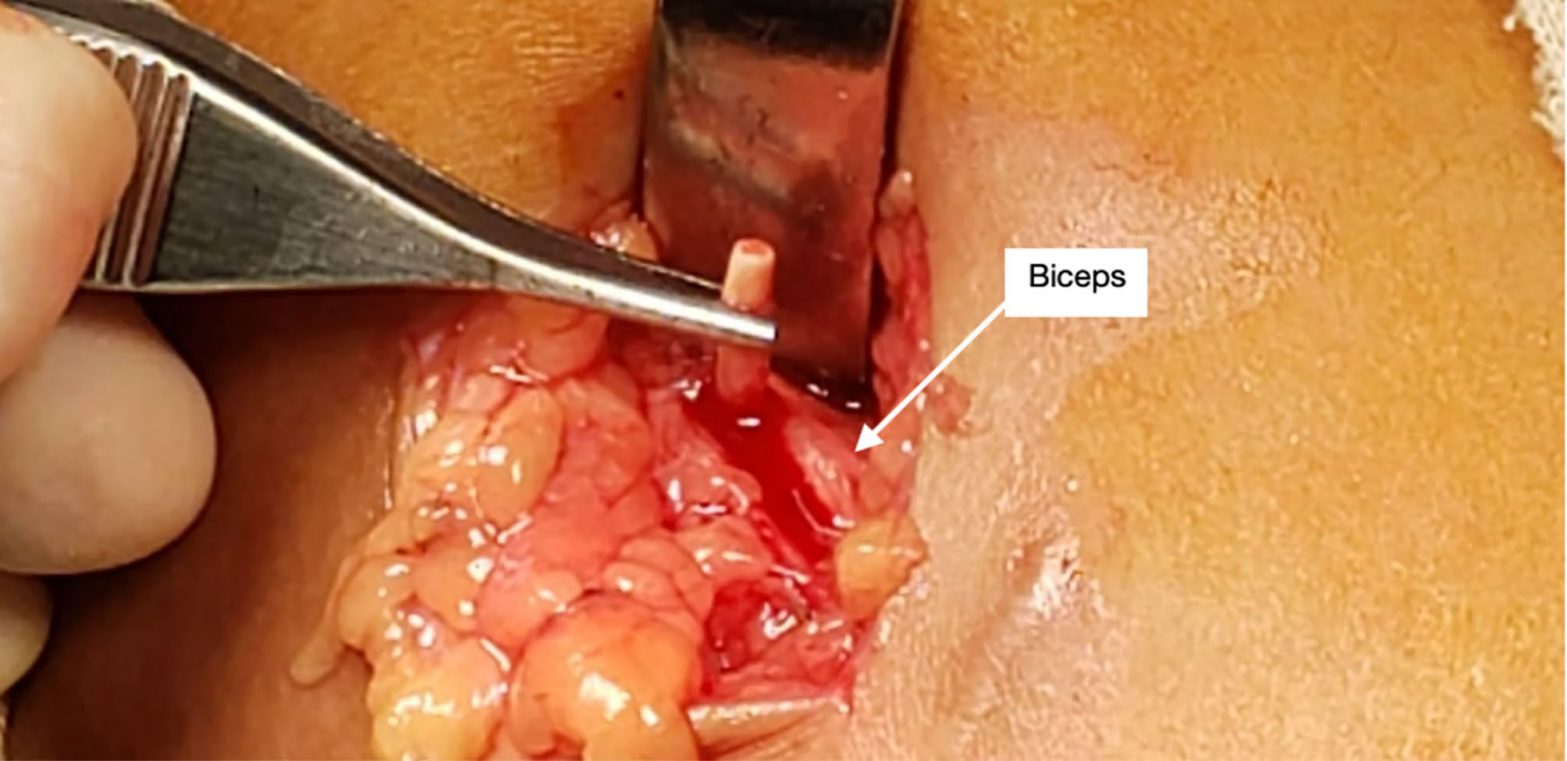
The rod was found intramuscularly, adjacent to the median nerve, and under the basilic vein

A 21-year-old woman presented at the Family Planning and Reproductive Health Clinic, King Chulalongkorn Memorial Hospital complaining of an unusual pain sensation at the surgical site associated with abnormal sensations and numbness in her left hand following the failure of contraceptive removal twice. The patient’s body mass index was 21.87 kg/m^2^. She experienced persistent spotting for approximately two and a half years after the insertion of an etonogestrel contraceptive implant, and early contraceptive implant removal was requested. Despite this, the implant had never been palpated by either the patient or healthcare providers. The patient underwent the first in-office implant removal at her local primary hospital, and the attempt was unsuccessful. During the procedure, the patient reported shooting pain at the surgical site that persisted after the procedure. Additionally, the patient reported abnormal sensations and numbness in her left hand without weakness. The patient later underwent a second in-office blind implant removal attempt, which failed and her pain and numbness worsened thereafter. One month after the procedure, the patient came with another removal request at the Family Planning and Reproductive Health Clinic, King Chulalongkorn Memorial Hospital. Physical examination showed a one-centimeter non-tendered surgical scar at the sulcus between the biceps and triceps muscles of the left upper arm. Hyperesthesia and a tingling sensation were observed around the surgical site. Strength testing did not reveal weakness in the median- and ulnar-innervated muscles. However, the implant could not be palpated. An experienced sonographer performed ultrasonography of the left upper arm to locate the implant; however, the implant could not be identified. Antero-posterior and lateral position X-ray scans of the left upper arm were performed and revealed that the distal end of the implant was 3 cm distant from the insertion scar as shown in Fig. 1. The patient was then referred to our orthopedic department. A 3-cm L-shaped incision was made in the left arm under fluoroscopic guidance and adhering to standard radiation protection measures for staff, including the use of lead aprons. The procedure was performed under general anesthesia by orthopedic hand specialist surgeons and the rod was successfully removed. Regarding the operative findings, the rod was found intramuscularly, 3 cm under the skin, adjacent to the median nerve, and under the basilic vein, as shown in Fig. 2. The procedure was performed without immediate complications. Postoperatively, the patient experienced mild peri-incisional pain. The unusual pain at the surgical site and together with abnormal sensations and numbness in her left hand gradually improved and completely resolved within a month. 

## Discussion

The case presented in this report is an example of an uncommon but possible adverse event that could occur because of the improper insertion and removal of contraceptive implants or implant migration over time, which is technically difficult to differentiate. Although the implant used was a Nexplanon implant, an applicator designed to control the direction and location of the implant, deep insertion or migration still occurred. An incorrect insertion technique can lead to improper placement of the implant and result in deep implantation, rendering the implant unpalpable [9]. Radiographs demonstrated that the rod was deeply embedded under the skin. In addition, the distal end of the rod was not located close to the scar, but 3 cm from the insertion scar. This may be caused by the migration of the rod over time or may occur as a result of blind attempts at removal causing iatrogenically deep implantation. In this case, we considered iatrogenic migration as the most likely cause of the patient’s symptom since the symptoms worsened after a blind in-office removal, even though the implant itself can migrate during its usage. Blind attempts at removal can cause the rod to migrate further and deeper from its original location. In this instance, such an attempt was the gravest mistake, potentially causing further injury by pushing the implant rod deeper into the structures beneath the arm. As noted in this patient, peripheral nerve and muscular damage caused by contraceptive implant removal manifested as worsening symptoms of pain, numbness, and abnormal sensations [5].

**Fig. 3:**
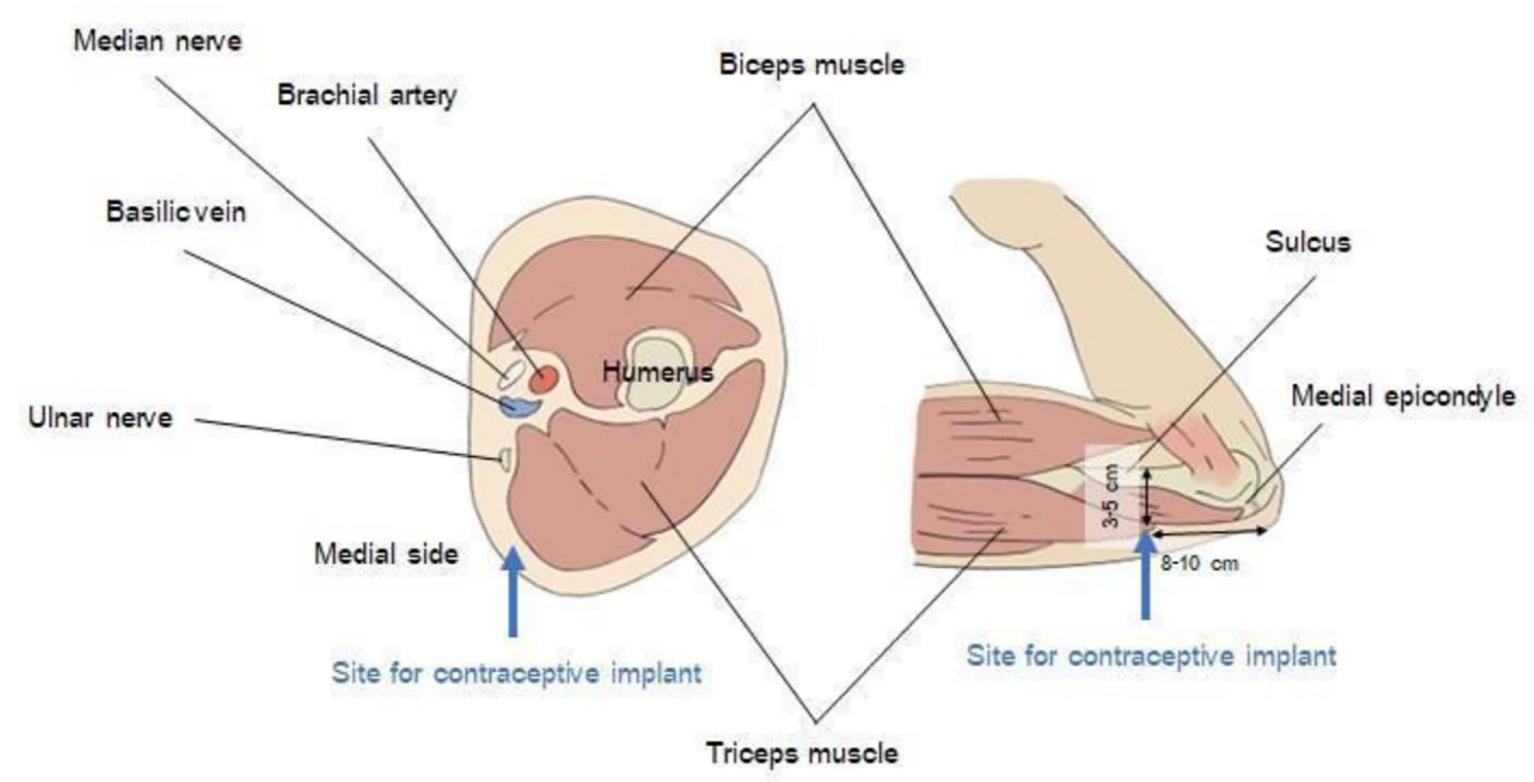
Proper location for contraceptive implant insertion [10]

The contraceptive implant should be placed subdermally just underneath the skin, 8–10 cm from the medial epicondyle. As shown in Fig. 3 [10], the implant should not be placed at the sulcus between the biceps and triceps muscles because of the location of the median nerve, ulnar nerve, brachial artery, and brachial vein [1]. In addition, it is crucial to keep the needle parallel to the skin surface during insertion [1]. In the present case, the implant was located in a muscle adjacent to the median nerve, close to the basilic vein. This may have resulted from an iatrogenic blind removal attempt, together with deeper insertion. As the implant rod was located within the muscle, it led to local irritation and, eventually, pain at the surgical site. Also, the rod, located adjacent to the median nerve, explained the patient’s symptoms of pain, abnormal sensations, and numbness in her hand. After careful removal without nerve injury, the patient’s symptoms resolved. Deep implantation can cause injury to neurovascular bundles, including the median nerve, ulnar nerve, medial cutaneous nerve of the forearm, and brachial artery [11]. There have been case reports of median nerve injury from blind removal of impalpable implants. Symptoms included thenar muscle weakness, dysesthesia, and paresthesia in the median nerve sensory distribution, which persisted for almost 2 years after implant removal [11]. Ulnar nerve injury can cause ulnar claw hand deformity and numbness in the ulnar nerve sensory distribution [5].

The contraceptive implant should always be palpable in the designated arm. Deeply placed implants or non-palpable implants should be precisely located and removed under adequate exploration to avoid further damage from distant migration or life-threatening complications such as pulmonary artery embolization or chronic neuropathy, which could cause significant morbidity [2]. Several methods can be used to locate the rod, such as radiography, computed tomography (CT), magnetic resonance imaging (MRI), and high-frequency linear ultrasound (10 MHz) [1]. In our case, we located the rod using the radiographs of the left upper arm. However, this method has the limitation of only providing a two-dimensional image, making it challenging to pinpoint the device’s exact location. Although ultrasound is a superior modality, we could not locate the rod using it. Our unfamiliarity with the procedure influenced this outcome. Blind removal of the rod without imaging guidance is associated with adverse outcomes [11, 12]. As evidence in our case, two attempts at blind removal led to heightened abnormal sensations, including increased pain and numbness. Several techniques for the removal of deeply placed or impalpable implants have been reported, all of which require the precise location of the implant to be identified using imaging [7, 13–15]. When surgically removing the implant, it should be done longitudinally, parallel to the arm, to prevent damage to adjacent structures. This case emphasizes the pivot of proper training for insertion and removal of implants. If the rod is not palpable, precise localization of the rod by imaging can help identify the site of the rod and prevent complications from implant migration. 

Abnormal sensations and pain aresigns of neurological and muscular involvement. If this occurs after the implantation procedure, the patient should be referred to a peripheral nerve or hand surgery specialist for prompt implant removal under adequate exposure and analgesia [2]. However, in-office removal should not be performed to prevent further injury to important structures in the arm owing to distant migration [2, 15]. In addition, implants can be localized intraoperatively under fluoroscopic guidance or ultrasonographic guidance, resulting in a more precise localization [3]. Fluoroscopy offers additional advantages, being a cost effective and readily accessible method that provides real-time imaging and is not operator-dependent. In this case, we opted for fluoroscopy over ultrasound, despite the latter being conducted by an experienced sonographer, due to our inability to pinpoint the location of the implant rod. Many studies on complications related to implant insertion and removal have suggested that proper training for implant insertion is crucial and should be routinely performed, even though the Nexplanon applicator is designed for simpler and easier insertion. Besides, complications associated with implant insertion and removal have led to lawsuits in several countries [4, 16].

## Conclusion

Although the etonogestrel implant is a contraceptive implant that acts as an applicator that limits the location of the implant, deeply and improperly placed implants can still occur. This case report is an example of the importance of referring patients with impalpable contraceptive rods to experienced specialists for implant removal, as doing so blindly can lead to further nerve damage from iatrogenic implant migration. Using precise imaging techniques, such as fluoroscopy or ultrasound, is crucial. It helps in determining the precise location of the implant, facilitating its safe removal.
